# LIMK1 Deficiency Disrupts Hippocampal–Cortical Memory Consolidation and Attenuates Trauma-Induced PTSD-like Behavior

**DOI:** 10.3390/biology14111560

**Published:** 2025-11-07

**Authors:** Xiangyu Yang, Zhengping Wu, Ziying Wang, Lihui Wang, Shuting Xia, Weidong Li, Guiqin He

**Affiliations:** 1 Key Laboratory for the Genetics of Developmental and Neuropsychiatric Disorders, Ministry of Education, Bio-X Institutes, Shanghai Jiao Tong University, Shanghai 200030, China; wangziying@sjtu.edu.cn (Z.W.); iwd@sjtu.edu.cn (W.L.); 2 Chongqing Research Institute, Shanghai Jiao Tong University, Chongqing 401151, China; 3 School of Electronic Science and Engineering, Nanjing University, Nanjing 210000, China; wzp_959@163.com; 4 Shanghai Key Laboratory of Psychotic Disorders, Brain Health Institute, National Center for Mental Disorders, Shanghai Mental Health Center, Shanghai Jiao Tong University School of Medicine, Shanghai 200030, China; lihui.wang@sjtu.edu.cn; 5 School of Psychology, Shanghai Jiao Tong University, Shanghai 200030, China; 6 Cancer Institute, Suzhou Medical College, Soochow University, Suzhou 215021, China; stxia@suda.edu.cn; 7 Global Institute of Future Technology, Shanghai Jiao Tong University, Shanghai 200240, China

**Keywords:** LIM-kinase, memory consolidation, hippocampal–cortical circuit, underwater trauma, PTSD-like behavior

## Abstract

Memory consolidation is the process by which newly acquired experiences are stabilized into long-term memory, involving coordinated cellular and network-level activity across brain regions such as the hippocampus and prefrontal cortex. Dysregulation of this process has been implicated in psychiatric disorders including post-traumatic stress disorder (PTSD), which is characterized by the over-consolidation of traumatic memories. LIM kinase 1 (LIMK1), a key regulator of synaptic plasticity, is believed to play an important role in memory consolidation across hippocampal–cortical circuits. In this study, we investigated the function of LIMK1 using *Limk1* knockout mice. Behavioral tests such as the novel object location memory task revealed significant memory impairments in knockout animals. In vivo recordings during sleep showed disrupted communication between the hippocampus and prefrontal cortex, suggesting impaired systems-level consolidation. Furthermore, in an underwater trauma exposure model, pharmacological inhibition of LIMK1 with LIMK-i3 alleviated trauma-induced behavioral abnormalities. These findings highlight LIMK1 as a critical mediator of hippocampal–cortical memory consolidation and provide experimental evidence that LIMK1 inhibition can modulate maladaptive memory processes associated with PTSD-like symptoms.

## Simple Summary

Our memories shape who we are, but how the brain turns short-term experiences into lasting memories is still not completely understood. This process, called memory consolidation, depends on the ability of brain cells to strengthen their connections and to communicate between brain regions during sleep. LIM kinase 1 (LIMK1) plays a key role in this process by regulating actin cytoskeleton dynamics, which are essential for maintaining dendritic spine structure and synaptic plasticity, the cellular basis of learning and memory. In this study, we investigated the role of LIMK1 in memory consolidation and emotional regulation using *Limk1* knockout mice, which were genetically engineered to lack the *Limk1* gene and therefore do not produce the LIMK1 protein. We found that these mice exhibited impaired formation of stable memories. We also found that blocking this protein with a drug reduced anxiety-like behaviors after stressful experiences. These findings suggest that LIM kinase 1 is essential for building long-term memories and that overactive memory consolidation may contribute to stress-related disorders such as post-traumatic stress disorder. Understanding how this protein works could lead to new ways to prevent or treat the harmful effects of traumatic memories and improve mental health in people who experience severe stress.

## 1. Introduction

Memory consolidation is a fundamental neurobiological process that transforms newly acquired experiences into long-term memories through coordinated cellular and systemic mechanisms [1,2]. At the cellular and synaptic levels, this process involves structural and functional modifications, including the expression of plasticity-related proteins and remodeling of the actin cytoskeleton [3,4]. Systemic consolidation refers to the gradual redistribution of information from the hippocampus to the neocortex, a process believed to depend on neural oscillatory activity, particularly during sleep, that facilitates hippocampal–cortical communication [5,6,7,8]. This process is thought to rely on neural oscillations generated during hippocampal–cortical coupling [9,10,11].

LIM-kinases, including LIMK1 and LIMK2, are key regulators of actin cytoskeletal dynamics and have been implicated in synaptic plasticity and memory formation [12,13]. *LIMK1*, located within the 7q11.23 chromosomal region linked to Williams Syndrome, is expressed in brain regions critical for cognitive processing such as the hippocampus and prefrontal cortex [14,15]. Structurally, LIMK1 is enriched at dendritic spines and synaptic compartments, positioning it to regulate spine morphology and synaptic signaling [16]. Functional studies have demonstrated that LIMK1 deficiency leads to altered spine morphology, impaired long-term potentiation, and deficits in long-term memory while sparing short-term memory [17,18,19]. Pharmacological inhibition of LIMK1 using specific inhibitors such as LIMK-i3 has been shown to influence multiple stages of memory processing, including acquisition, consolidation, retrieval, and reconsolidation [20,21].

The mechanisms of memory consolidation are highly relevant to understanding post-traumatic stress disorder (PTSD), a chronic psychiatric condition that can arise following exposure to traumatic events [22]. PTSD is characterized by hyperactivity and dysfunction in brain regions involved in fear learning and memory, including the hippocampus, prefrontal cortex, and amygdala [22,23,24]. This dysregulation may facilitate the formation and persistence of maladaptive memories, contributing to symptoms such as intrusive recollections, hyperarousal, and avoidance behaviors [25,26]. Over-consolidation of traumatic memories, wherein emotionally salient memories become excessively stabilized and resistant to extinction, has been proposed as a key mechanism underlying PTSD symptomatology [27,28]. However, the molecular pathways contributing to this process remain incompletely understood.

The present study investigates the role of LIMK1 in memory consolidation and its implications for PTSD pathogenesis. Using *Limk1* knockout (KO) mice, we evaluated memory performance through behavioral paradigms including the object location recognition (OLR) test, which probes spatial memory encoding and retrieval [29]. We further examined sleep-associated memory consolidation between the hippocampus and mPFC through in vivo neural recordings. To assess the functional relevance of LIMK1 in trauma-related memory processing, we utilized an underwater trauma model and evaluated the effect of LIMK inhibition with LIMK-i3. Our results indicate that LIMK1 is critical for memory consolidation and is associated with changes in hippocampal–cortical network activity, although direct causality at the level of network coordination remains to be established. Moreover, pharmacological inhibition of LIMK1 alleviated PTSD-like behaviors following trauma exposure, thereby highlighting LIMK1 as a potential therapeutic target for trauma-related disorders such as PTSD.

## 2. Materials and Methods

### 2.1. Animals

All animal experiments were approved by Shanghai Mental Health Center Animal Care and Use Committees and were designed to minimize suffering and the number of animals used. Male mice aged 6–8 weeks and weighing 20–25 g were used for all experiments. *Limk1* KO (*n* = 28) mice and their corresponding wild-type (WT, *n* = 27) littermates were generated by intercrossing heterozygous breeders. C57BL/6J WT mice (*n* = 184) used for experimental model establishment and pharmacological intervention were obtained from GemPharmatech (Nanjing, China). Mice were housed five per cage under controlled photoperiods and temperature (12 h reversed light/dark cycle at 21 ± 2 °C) with access to standard food and water. Animals were acclimated to their cages for at least one week before acclimating to the test devices began.

### 2.2. Cannula Implantation and Drug Micro-Infusion

Cannula implantation was performed only in the pharmacological intervention experiments. Mice were deeply anesthetized by an i.p. injection of pentobarbital sodium (1%, 10 μL/g) and 30-gauge guide cannula (RWD, Shenzhen, China) bilaterally implanted in the brain targeting the dorsal hippocampus (−2.0 mm AP, ±1.5 mm ML, −1.4 mm DV) and mPFC (+1.8 mm AP, ±0.2 mm ML, 1.7 mm DV). Before the behavioral produces, animals were allowed to recover for at least 5 days. Micro-infusions into the target region (vehicle or LIMK-i3 200 μM in 1 μL) were made at a rate of 0.2 μL/min. The injection cannula was left in place for 5 min after micro-infusion and the mice were then returned to their home cages. Following the behavioral experiments, subjects were sacrificed and their brains were dissected and stained to verify for cannula position. Only mice with a cannula tip within the boundaries of the target region were included in the data analysis.

### 2.3. Object Location Recognition Task

Object location recognition was conducted using a modified version of the method described by Yuan et al. [30]. Briefly, mice were handled 3–5 min for 3 days before training. And then, the mice were allowed to habituate to the experimental apparatus (blue square open field, 50 × 50 × 50 cm; a white card (40 × 15 cm) on one wall served as a visual reference cue) for 15 min once a day for 3 consecutive days. During encoding, mice were placed into the apparatus with two identical objects placed in two adjacent corners and were allowed to explore for 15 min. The tests took place 2 h later for short-term memory or 24 h for long-term memory. During the test, one of the objects was displaced to the opposite corner and the animal was allowed to explore the experimental apparatus for 5 min. Video tracking software (Ethovision XT 11.5, Noldus, Wageningen, The Netherlands) was used to measure the amount of time the mouse spent exploring the object. The discrimination index (DI) was calculated using the following formula: DI = (time exploring the displaced object − time exploring the nondisplaced object)/total time exploring object × 100.

In the comparative experiments between WT (*n* = 12) and KO (*n* =12) mice, long-term and short-term OLR tests were conducted one week apart, each performed in open fields with distinct contextual cues to minimize environmental memory interference. The objects used in the OLR tasks were also replaced between sessions to prevent object-specific familiarity effects.

In the pharmacological intervention experiments, C57BL/6J WT mice were implanted with bilateral cannulas targeting either the mPFC (*n* = 23) or hippocampal CA1 region (*n* = 27). Cannula-implanted mice were randomly assigned to either the drug-treatment or control group. One week after the long-term OLR test, the animals were re-randomized into two groups for the short-term behavioral test, which was carried out in open fields with different contextual backgrounds and replaced objects to minimize potential environmental and object-related memory bias. Cannula-implanted mice received micro-infusion of the vehicle or LIMK-i3 immediately after the encoding phase. Mice with detached cannulas or incorrectly verified implantation sites were excluded from the final data analysis.

### 2.4. EEG and EMG Electrode Implantation

A total of 19 male mice (9 WT mice and 10 *Limk1* KO mice) were used for sleep recording experiments. Mice were anesthetized with pentobarbital sodium (1%, 10 μL/g, i.p.) and placed in a stereotaxic apparatus (RWD, Shenzhen, China). EEG (electroencephalogram) and EMG (electromyogram) electrodes were surgically implanted for sleep recording. EEG electrodes (stainless steel screws) were implanted epidurally over the parietal cortex (AP: +1.0 mm; ML: +1.0 mm) and the cerebellum. EMG electrodes (stainless steel wires) were bilaterally inserted into the neck muscles.

After surgery, mice were allowed to recover and acclimate to the sleep recording chamber for one week. The animals were then connected to the Neurokey recording system (Greathink Tech, Nanjing, China) for EEG/EMG monitoring. After a 24 h habituation period to the recording setup, each mouse was recorded continuously for 48 h under a 12 h light/12 h dark cycle with ad libitum access to food and water.

### 2.5. Sleep Scoring and Quantification

Scoring was first done semiautomatically and then visually inspected based on EEG-EMG waveforms and power spectra, with corrections applied as needed. All scoring was performed by an investigator blind to the experimental group. Wakefulness was defined by low-amplitude, high-frequency EEG and high EMG tone; NREM sleep by high-amplitude, low-frequency EEG (0.5–4 Hz) and low EMG activity; and REM sleep by dominant theta rhythm (6–9 Hz) with muscle atonia. EEG power spectra were analyzed using fast Fourier transformation and expressed relative to the total power of the same stage. Sleep parameters, including total sleep time, wake, NREM, and REM sleep duration were quantified separately for the light and dark phases.

### 2.6. In Vivo Electrophysiology

In vivo electrode implantation and recording were performed as described in previous studies [31]. Briefly, WT and *Limk1* KO mice (*n* = 6 per genotype) were anesthetized with pentobarbital sodium (1%, 10 uL/g, i.p.) and placed in a stereotaxic instrument (RWD, Shenzhen, China). Eye ointment was applied to prevent corneal drying and a heat pad was used to hold body temperature at 37 °C. A small craniotomy hole was made using a dental drill. Then the mice were implanted with custom electrodes targeting the hippocampus (AP: −1.94 mm; ML: +1.50 mm; DV: −1.50 mm) and prefrontal cortex (AP: +1.70 mm; ML: +0.25 mm; DV: −1.70 mm). Every electrode consisted of four independently adjustable nichrome probes (35 μm for diameter) arranged in two rows and insulating barrier plated to reach an impedance of 200–250 kΩ. Two wires attached to stainless steel screws placed on the posterior head were used as ground and reference. After surgery, the mouse received carprofen (5 mg/kg) for postoperative analgesia and was allowed to recover for one week.

Following recovery, mice performed the object location recognition (OLR) behavioral paradigm, during which neuronal activity was recorded for 2 h both before and after the encoding phase. Neuronal signals were recorded at 30 kHz using the Neurolego system (Greathink tech, Nanjing, China). Local field potentials were band pass filtered at 0.5–300 Hz and stored with 3000 Hz sampling rate. After the LFP recording, a current (100 μA, 10 s) passed between two branches of electrodes was used to mark the end of the electrode tract. Electrode tip positions were verified by Nissl staining.

### 2.7. Electrophysiological Analysis

Data were analyzed using MatlabR2020b (The MathWorks Inc., Natick, MA, USA) with FMA-Toolbox (http://fmatoolbox.sourceforge.net, accessed on 1 October 2019). In offline analysis, wave detection was performed with Neurospace (Greathink tech, Nanjing, China) using the algorithm developed by Maingret [8]. For ripple wave detection, the raw data of LFP was band filtered at 100–250 Hz, squared, low-pass filtered and normalized. Ripple waves were identified whenever signal surpass the threshold, peaks > 3 times the standard deviation cross all filtered signal points, for the duration at least 30 ms but no longer than 100 ms. The up-ward and down-ward threshold crossings were defined as the beginning and ending of the ripple event.

The detection of delta waves followed same procedure. The raw LFP signals from mPFC were band filtered at 0.1–5 Hz frequency range. Delta waves respond to sequences lasting between 150 ms and 500 ms with the peak larger than 2 times standard deviation across all filtered signal points.

For spindle detection, the LFP signals recorded in the mPFC was band-pass filtered (9–17 Hz), smoothed, and normalized. Spindles were defined as waves where signal stayed above 2.5 times standard deviation for more than 500 ms and peaked at above 5 times standard deviation.

All coupling analyses were performed specifically during sleep. Delta-spindle (D-S) coupling was defined as sequences where spindle wave peaks occurred between 100 ms and 1300 ms after delta wave peaks. Ripple-delta (R-D) coupling was defined as sequences where delta peaks occurred between 50 ms and 250 ms after ripple peaks. Ripple-delta-spindle (R-D-S) sequences corresponded to the conjunction of these electrical events.

### 2.8. Water-Associated Zero Maze (WAZM)

The WAZM is an adaptation of the traditional elevated zero maze, incorporating both wet and dry environments [32]. This apparatus enables the formation of an association of the maze with an underwater trauma (UWT), and by that, the assessment of behaviors during re-exposure to the context which immediately precedes a traumatic experience. In the experimental procedure, mice are initially acclimated to the testing room for a duration of 2 min. Subsequently, they were placed into one of the open arms facing a closed part of the apparatus. Mice were allowed to explore the arena for a 5 min session. During this time, mice’s behavior was tracked, recorded and analyzed by the video tracking software (Ethovision XT 11.5, Noldus). Behavioral measures included the time spent in the open arms, distance traveled in the open arms, distance traveled in the closed arms.

### 2.9. Underwater Trauma and Re-Exposure

Animals were randomly assigned to either control or UWT conditions. The UWT stress was conducted immediately after the fourth habituation. UWT mice were lifted from the dry arms and placed in the aquatic center of the WAZM, which contained water at 22 °C ± 2 °C and was 50 cm deep. They were submerged under water for 30 s using a special plastic net (diameter 20 cm). After the procedure, the animals were briefly dried and placed on an open arm for 5 min before being returned to their housing cages. Thirty days later, the mice were re-exposed to the WAZM for 5 min to assess behaviors induced by the traumatic experience.

For the pharmacological intervention experiments, C57BL/6J WT mice were implanted with bilateral guide cannula targeting either the mPFC (*n* = 37) or hippocampal CA1 (*n* = 34) at least 5 days prior to behavioral procedures to allow full recovery. The mice were randomly assigned to three groups: Control Vehicle, UWT Vehicle, and UWT LIMK-i3. Micro-infusions into the target region (vehicle or LIMK-i3, 200 μM in 1 μL) were performed at a rate of 0.2 μL/min immediately following the 5 min open arm exposure.

### 2.10. Western Blot

To examine the temporal dynamics of LIMK1 and Cofilin activation following underwater trauma (UWT), C57BL/6J WT mice were sacrificed at 0.5 h, 2 h, and 6 h after UWT exposure for brain sample collection. Each experimental group consisted of four mice. Corresponding control mice were processed at the same time points without undergoing UWT. Protein lysates from the mPFC and hippocampus were extracted using RIPA lysis buffer supplemented with 1% protease and phosphatase inhibitor (Roche, Basel, Switzerland). Centrifugation at 14,000× *g* for 10 min at 4 °C was performed to remove debris. The protein samples were separated on SDS-PAGE and transferred onto a PVDF membrane. After blocking with 5% nonfat dry milk in 1 × TBST buffer for 1 h at room temperature, the membranes were incubated overnight at 4 °C with primary antibodies: Cofilin (Cell Signaling Technology, Danvers, MA, USA, #5175S, 1:2000), p-Cofilin (Santa Cruz, Santa Cruz, CA, USA, sc-271921, 1:1000), which is specific for detecting Cofilin phosphorylated at Serine 3, a site directly targeted and inactivated by LIMK1; LIMK1 (Cell Signaling Technology, #3842S, 1:2000), p-LIMK1 (Affinity, Cincinnati, OH, USA, AF3345, 1:1000), which specifically detects LIMK1 phosphorylated at Threonine 508, a modification associated with its activation; GAPDH (Proteintech, Chicago, IL, USA, 60004-1-Ig, 1:5000). Subsequently, the membranes were washed three times with 1 × TBST buffer and incubated with HRP-conjugated secondary antibodies: anti-mouse (Proteintech, SA00001-1, 1:5000) and anti-rabbit (Proteintech, SA00001-2, 1:5000) for 1 h at room temperature. Finally, protein bands were visualized using an HRP substrate (Millipore, Burlington, MA, USA) and detected with an iBright FL1000 imaging system (Invitrogen, Carlsbad, CA, USA). The PVDF membrane was first used to detect phosphorylated Cofilin (p-Cofilin) and phosphorylated LIMK1 (p-LIMK1). Phosphorylated antibodies were then removed using an antibody stripping buffer (CW0056M, CWBIO, Beijing, China), after which the membrane was separately incubated with antibodies specific to total Cofilin and total LIMK1.

### 2.11. Statistical Analysis

In all experiments, the experimenters were blind to the genotype and treatment history of the mice. The data were analyzed using Student’s *t*-test, one-way ANOVA, or two-way ANOVA, as appropriate. Post hoc between-group comparisons were conducted using Tukey’s multiple comparisons test or Bonferroni’s post hoc test. Values were presented as means ± standard errors of the means (SEM). Results with *p* < 0.05 were regarded as statistically significant (indicated with asterisk in summary graphs).

## 3. Results

### 3.1. LIMK1 Deficiency Significantly Prevents Memory Consolidation

**Figure 1:**
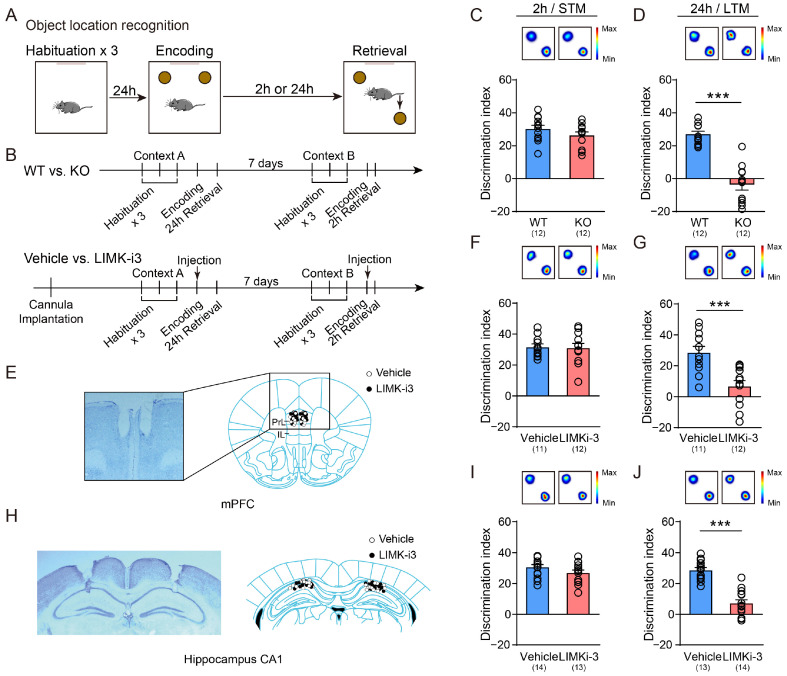
LIMK1 deficiency or functional inhibition impair long-term memory for object location. (**A**) Schematic representation of the OLR task, with brown circles representing two similar objects and a black arrow indicating a moved familiar object. Visual cues were on the one side of the training arena wall. (**B**) OLR experimental design: upper, WT vs. *Limk1* KO; lower, post-encoding LIMK-i3 vs. vehicle in mPFC or hippocampus. (**C**) Results from the 2 h short-term memory retention test showing no significant difference in performance between LIMK1 deficient and wild-type mice. (**D**) Results from the 24 h long-term memory retention test of LIMK1 deficient and wild-type mice. (**E**) Illustration showing the location of LIMK-i3 micro-infusion cannula tips in the mPFC. White dots: Vehicle; Black dots: LIMK-i3. (**F**,**G**) Performance in the short-term and long-term OLR task with LIMK activity inhibition in mPFC. (**H**) Illustration showing the location of LIMK-i3 micro-infusion cannula tips in the hippocampus CA1. White dots: Vehicle; Black dots: LIMK-i3. (**I**,**J**) Performance in the short-term and long-term OLR task with LIMK1 activity inhibition in hippocampus CA1. Data are mean ± SEM. Heat maps indicate time exploring the objects. Statistical analysis was performed using Student’s *t*-test. Circles represent individual data points. The number of samples (*n*) for each group is shown in parentheses in the figure. *** *p* < 0.001.

In the object location recognition (OLR) test (Figure 1A,B upper panel), LIMK1 deficiency did not alter recognition performance in the short-term memory task (Figure 1C, Student’s *t*-test). However, LIMK1-deficient mice exhibited significantly impaired performance in the long-term OLR test (Figure 1D, Student’s *t*-test, *p* < 0.001).

Next, cannulas were implanted into the mPFC or hippocampus, and LIMK1 activity was inhibited by LIMK inhibitor (LIMK-i3) injection immediately after encoding (Figure 1B, lower panel). Nissl-stained coronal sections that show the representative cannula placement in the mPFC (Figure 1E) and dorsal hippocampus (Figure 1H), respectively. Memory performance was assessed 2 h after encoding to evaluate short-term memory and 24 h after encoding to evaluate long-term memory. Our results showed that there was no difference in short-term memory performance between the vehicle-treated and LIMK-i3-treated groups in the mPFC (Figure 1F, Student’s *t*-test). However, long-term memory was impaired in the mPFC LIMK-i3-treated group, as evidenced by a significant decrease in long-term memory performance compared to the vehicle group (Figure 1G, Student’s *t*-test, *p* < 0.001). We also found that LIMK-i3 infusion into the hippocampus did not affect short-term memory (Figure 1I, Student’s *t*-test) but impaired long-term memory expression (Figure 1J, Student’s *t*-test, *p* < 0.001). However, neither global knockout nor region-specific inhibition of LIMK1 produced significant effects on baseline locomotor activity in mice (Supplementary Figure S1, Student’s *t*-test).

### 3.2. Sleep/Wake Time and Architecture of Limk1 KO Mice

**Figure 2:**
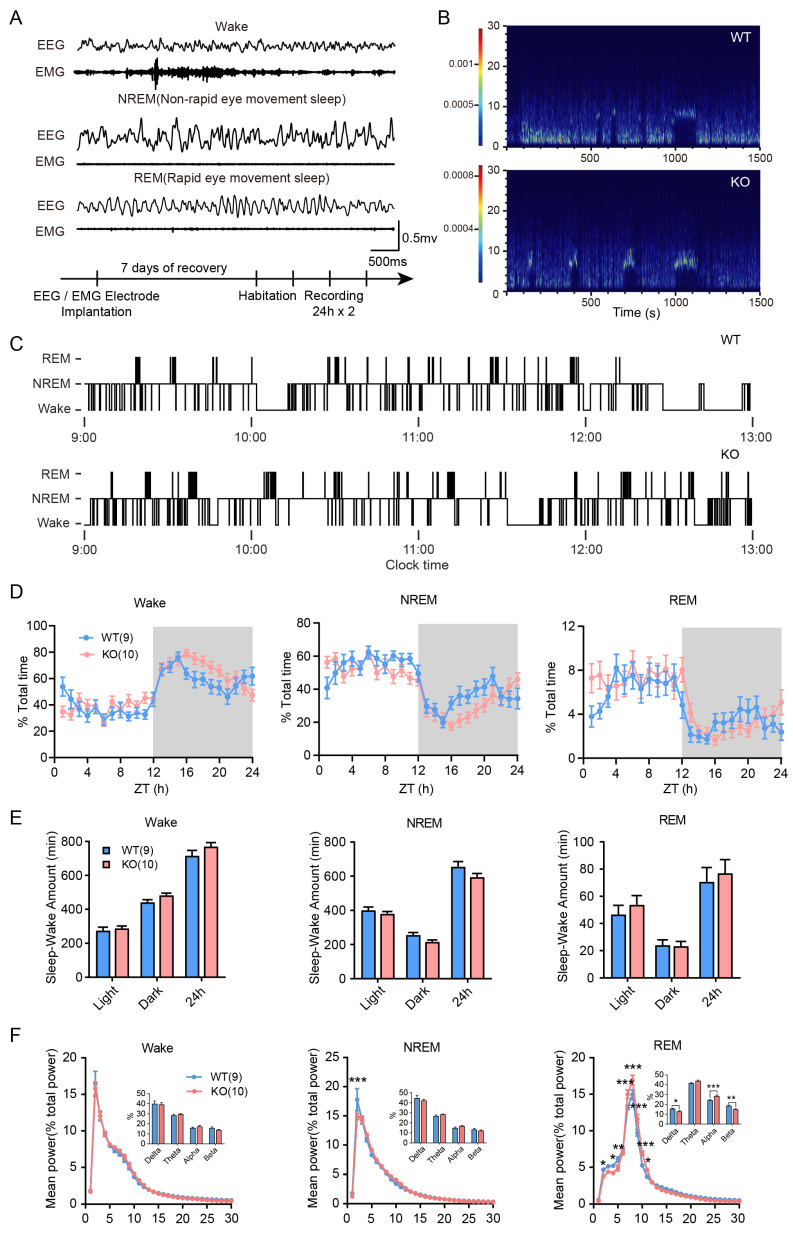
*Limk1* KO mice exhibit normal sleep structure with EEG changes at specific Sleep–Wake stages. (**A**) Representative EEG and EMG waveforms during different sleep–wake stages and schematic representation of the experimental procedures for EEG/EMG recording. (**B**) EEG power spectra of WT (top) and *Limk1* KO (bottom) mice. (**C**) Hypnograms over a 4 h period, showing comparable sleep episodes in *Limk1* KO mice. (**D**) Time course of wakefulness, NREM sleep, and REM sleep during the dark and light phases. (**E**) Comparison of total sleep–wake amounts between WT and KO mice. (**F**) EEG power density curves for wakefulness, NREM, and REM sleep, with quantitative analysis of power across different frequency bands. Data are presented as mean ± SEM. Statistical analysis was performed using Two-way ANOVA followed by Bonferroni’s post hoc test. *n* = 9 for WT group. *n* = 10 for KO group, each mouse was recorded for 48 h. The number of samples (*n*) for each group is also shown in parentheses in the figure. * *p* < 0.05, ** *p* < 0.01, *** *p* < 0.001.

Due to the important role that sleep plays in the process of memory consolidation, we conducted a comparative analysis of the sleep state of *Limk1* KO mice through in vivo recording. As shown in Figure 2A, we were able to differentiate the mice’s states through EEG and EMG recordings. Through visual observation of the spectrogram (Figure 2B) and hypnograms (Figure 2C), we did not find obvious differences in sleep structure between WT and KO mice. Subsequently, we further analyzed the sleep–wake cycles in different state. In terms of the proportion of sleep states, there was no significant difference in the duration of sleep states between WT and KO mice (Figure 2D,E, two-way ANOVA followed by Bonferroni’s post hoc test, 2D, F_wake_ (1, 36) = 1.870; F_NREM_ (1, 36) = 2.415; F_REM_ (1, 36) = 0.2281; 2E, F_wake_ (1, 108) = 0.4820; F_NREM_ (1, 108) = 6.177; F_REM_ (1, 108) = 4.354). However, KO mice showed significant differences in the delta frequency (*p* < 0.001) band of non-rapid eye movement sleep and the delta (*p* < 0.05), alpha (*p <* 0.01) and beta (*p* < 0.01) frequency bands of rapid eye movement sleep (Figure 2F, two-way ANOVA followed by Bonferroni’s post hoc test, Student’s *t*-test for inter-frequency band comparison, F_wake_ (1, 1650) = 1.231 × 10^−14^; F_NREM_ (1, 1650) = 8.996 × 10^−13^; F_REM_ (1, 1650) = 3.618 × 10^−14^).

### 3.3. Mice Deficient in LIMK1 Show Impaired Coupling Between the mPFC and Hippocampus

**Figure 3:**
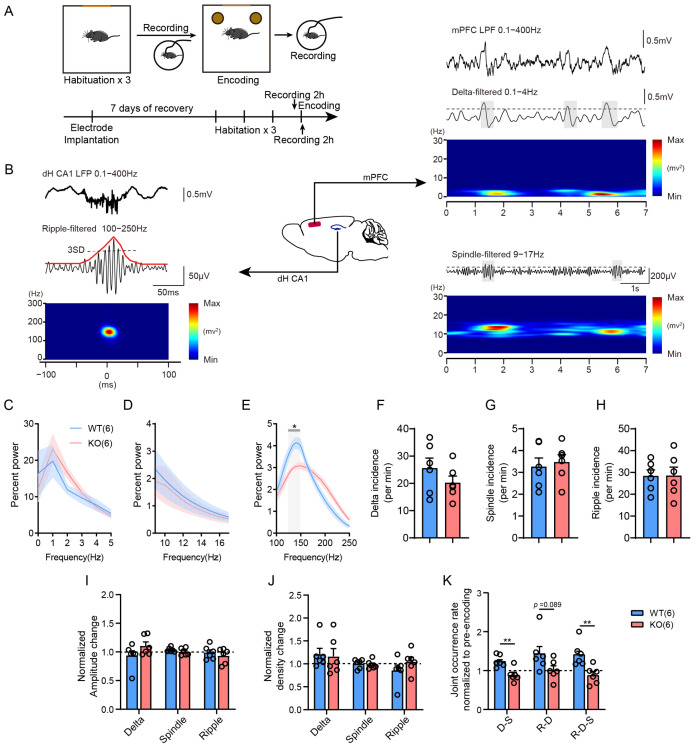
Comparative analysis of LFP dynamics in WT and *Limk1* KO mice after Object Location Recognition task. (**A**) Schematic of the Object Location Recognition task and in vivo recording. (**B**) Example traces of LFPs recorded in the CA1 (left: raw and ripple-band-filtered) and mPFC (right: raw, delta-band-filtered, and spindle-band-filtered) during a typical recording session in one mouse. Gray and red lines indicate the onset and offset of identified ripples (100–250 Hz), delta waves (0.1–4 Hz), and spindles (9–17 Hz). (**C**) Delta power density in WT and *Limk1* KO mice. (**D**) Spindle power density in WT and *Limk1* KO mice. (**E**) Ripple power density in WT and *Limk1* KO mice. (**F**) Incidence of delta waves recorded in the mPFC of WT and *Limk1* KO mice. (**G**) Incidence of spindles recorded in the mPFC of WT and *Limk1* KO mice. (**H**) Incidence of ripples recorded in the dorsal hippocampus CA1 of WT and *Limk1* KO mice. (**I**) Normalized amplitude of delta, spindle, and ripple in WT and *Limk1* KO mice. (**J**) Normalized density of delta, spindle, and ripple in WT and *Limk1* KO mice. (**K**) Joint occurrence rate of delta, spindle, and ripple coupling in WT and *Limk1* KO mice. Data are mean ± SEM. Statistical analysis was performed using Two-way ANOVA followed by Bonferroni’s post hoc test or Student’s *t*-test. Circles represent individual data points. *n* = 6 for each group. The number of samples (*n*) for each group is also shown in parentheses in the figure. * *p* < 0.05, ** *p* < 0.01.

Dual electrodes were implanted in the hippocampal CA1 region and the prefrontal cortex to record local field potentials (LFPs) in mice before and after memory encoding (Figure 3A,B). Our findings indicate that LIMK1 deficiency did not significantly alter delta power density (Figure 3C, two-way ANOVA followed by Bonferroni’s post hoc test, F (1, 10) = 0.1976) or spindle power density (Figure 3D, two-way ANOVA followed by Bonferroni’s post hoc test, F (1, 10) = 0.08982). However, *Limk1* KO mice exhibited a peak shift and decreased in peak ripple power density (Figure 3E, two-way ANOVA followed by Bonferroni’s post hoc test, F (1, 10) = 0.1112, post hoc test, 125–150 Hz, *p* < 0.05). Measures such as incidence (Figure 3F–H, Student’s *t*-test), normalized amplitude (Figure 3I, Student’s *t*-test), and density (Figure 3J, Student’s *t*-test) of oscillations were unaffected. Importantly, the coupling rate between prefrontal cortical delta waves and spindle oscillations (D–S), as well as among hippocampal sharp wave ripples, prefrontal cortical delta waves, and spindle oscillations (R–D–S), significantly increased after encoding in WT mice but was impaired in *Limk1* KO mice (Figure 3K, Student’s *t*-test, DS: *p* < 0.01; R-D-S: *p* < 0.01; Supplementary Figure S2).

### 3.4. Hippocampus and mPFC LIMK1/Cofilin Are Activated After Underwater Trauma to Develop PTSD-like Behavior

**Figure 4:**
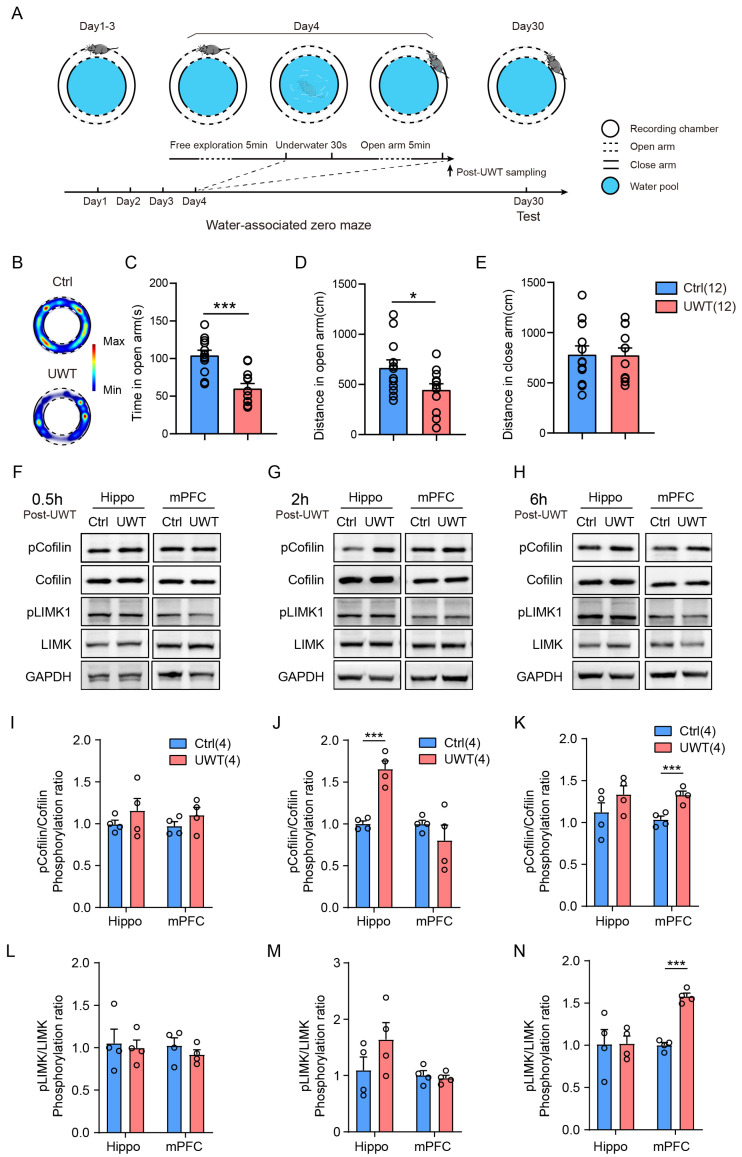
Activation of LIMK1/Cofilin signaling in the hippocampus and mPFC following underwater trauma stress. (**A**) Diagram illustrating the experimental procedures. (**B**) Representative activity heat maps of control (Ctrl) and underwater trauma (UWT) mice during the water-associated zero maze test. (**C**) Time spent exploring the open arm of the water-associated zero maze. (**D**) Distance traveled in the open arm of the water-associated zero maze. (**E**) Distance traveled in the closed arm of the water-associated zero maze. (**F**–**H**) Representative Western blot images of hippocampus and mPFC lysates at 0.5, 2, and 6 h post-underwater trauma. (**I**–**K**) Ratios of phosphorylated Cofilin in hippocampus and mPFC lysates at 0.5, 2, and 6 h post-underwater trauma. (**L**–**N**) Ratios of phosphorylated LIMK1 in hippocampus and mPFC lysates at 0.5, 2, and 6 h post-underwater trauma. Data are mean ± SEM. Statistical analysis was performed using Student’s *t*-test. Circles represent individual data points. The number of samples (*n*) for each group is also shown in parentheses in the figure. * *p* < 0.05, *** *p* < 0.001.

In the underwater trauma (UWT) stress model (Figure 4A), mice exhibited significantly reduced exploration time and a marked decrease in movement distance in the open arms of the zero maze (Figure 4B–D, Student’s *t*-test, C: *p* < 0.001; D: *p* < 0.05). In contrast, the movement distance in the closed arms did not show significant changes (Figure 4E, Student’s *t*-test). Then, we measured the activity of LIMK1 and its downstream target protein Cofilin in the hippocampus and medial prefrontal cortex (mPFC) at 0.5, 2, and 6 h post-modeling by using specific phosphorylated antibodies. An anti-phospho-LIMK1 (Thr508) antibody was used as a marker of LIMK1 activation, and an anti-phospho-Cofilin (Ser3) antibody specifically detects Cofilin phosphorylated at Serine 3, the site directly targeted and inactivated by LIMK1 [33,34,35]. As shown in Figure 4F,I,L, there was no significant difference in the phosphorylation of Cofilin and LIMK1 between the control and UWT groups 0.5 h post-trauma. However, at 2 h post-trauma, there was an increase in the phosphorylation of Cofilin and LIMK1 in the hippocampus (Figure 4G,J,M, Student’s *t*-test, *p* < 0.001). This increase was observed in the mPFC 6 h post-trauma (Figure 4H,K,N, Student’s *t*-test, *p* < 0.001). However, no significant differences were observed in the basal expression levels of LIMK1 and its downstream effector Cofilin (Supplementary Figure S3).

### 3.5. Hippocampus and mPFC LIMK1 Inhibition During Memory Consolidation in Reducing Trauma-Induced PTSD-like Behavior

**Figure 5:**
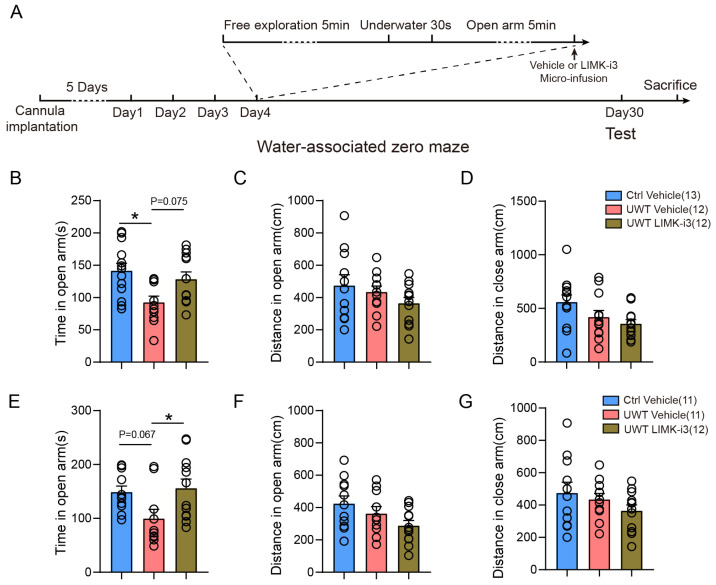
LIMK-i3 inhibition ameliorates UWT-induced PTSD-like behavior. (**A**) Schematic representation of the experimental procedures for LIMK1 inhibition in the UWT test. (**B**) Time spent in the open arm of the water-associated zero maze by mice subjected to mPFC LIMK1 inhibition. (**C**) Distance traveled in the open arm by mice subjected to mPFC LIMK1 inhibition. (**D**) Distance traveled in the closed arm by mice subjected to mPFC LIMK1 inhibition. (**E**) Time spent in the open arm of the water-associated zero maze by mice subjected to hippocampal LIMK1 inhibition. (**F**) Distance traveled in the open arm by mice subjected to hippocampal LIMK1 inhibition. (**G**) Distance traveled in the closed arm by mice subjected to hippocampal LIMK1 inhibition. Statistical analysis was performed using One-way ANOVA followed by Tukey’s multiple comparisons test. Circles represent individual data points. The number of samples (*n*) for each group is shown in parentheses in the figure. Data are mean ± SEM. * *p* < 0.05.

LIMK1 deficiency was associated with disrupted memory consolidation and impaired long-term memory formation. To further examine this effect, cannulas were implanted into the mPFC or hippocampus of mice, and LIMK1 activity was immediately inhibited using LIMK-i3 injection following underwater trauma. PTSD-like behavior was evaluated 30 days post-inhibitor injection (Figure 5A). Our findings showed that, compared to the vehicle-injection group, LIMK-i3 administration into the mPFC led to a moderate increase in open-arm exploration time, while hippocampal injection resulted in a significant increase (Figure 5B,E, One-way ANOVA followed by Tukey’s multiple comparisons test, Figure 5B: F(2,33) = 5.254, Ctrl Vehicle vs. UWT Vehicle, *p* < 0.05; Figure 5E: F(2,30) = 3.862, UWT Vehicle vs. UWT LIMK-i3, *p* < 0.05). These results suggest that LIMK1 inhibition impairs the consolidation and formation of trauma-related memories. However, both the mPFC (Figure 5C,D, One-way ANOVA followed by Tukey’s multiple comparisons test, Figure 5C: F(2,33) = 2.929; Figure 5D, F(2,33) = 4.101) and hippocampus (Figure 5F,G, One-way ANOVA followed by Tukey’s multiple comparisons test, Figure 5F: F(2,30) = 2.979; Figure 5G: F(2,30) = 1.590) LIMK-i3-treated groups exhibited decreased movement distances in both the open and closed arms.

## 4. Discussion

Memory consolidation is a crucial process involving the transformation of short-term memory into long-term memory, which entails changes in synaptic structures and functional connectivity in neural circuits [36,37,38]. As a cell cytoskeleton regulatory protein, LIMK1 directly participates in the modulation of synaptic plasticity [15,18,39]. Previous studies have reported that its absence can lead to abnormal long-term potentiation (LTP) and hinder long-term memory formation [17]. Pharmacological inhibition of hippocampal LIMK with LIMK-i3 impairs memory acquisition, consolidation, retrieval, and reconsolidation, but not extinction, in contextual fear conditioning [21]. Consistent with these findings, our study demonstrated that both genetic deletion and pharmacological inhibition of LIMK1 disrupted long-term memory formation in the object location recognition paradigm, without affecting short-term memory (Figure 1). Although the involvement of LIMK1 in memory has been validated at multiple levels, the influence of its absence on circuit-level effects has not been extensively studied.

It is well-known that memory consolidation is closely related to the transformation from short-term to long-term memory and relies on coordinated interplay among multiple brain regions during sleep [40,41,42,43]. We first analyzed the sleep–wake architecture of *Limk1* knockout mice (Figure 2A,B). The results showed no significant differences in the distribution preferences and duration of NREM and REM stages (Figure 2C–E), indicating that LIMK1 loss does not overtly disrupt baseline sleep architecture. This finding is important, as it suggests that subsequent neural differences are not secondary to altered sleep quantity or structure.

However, analysis of the power spectral density of brainwaves during different states revealed notable differences in the delta frequency band of non-rapid eye movement (NREM) sleep and the delta, alpha, and beta frequency bands of rapid eye movement (REM) sleep (Figure 2F). These results suggest that while the overall sleep structure remains normal, LIMK1 deficiency leads to changes in sleep related oscillatory activity, potentially reflecting altered neural network dynamics.

Interestingly, our analysis of brain rhythms revealed that *Limk1* KO had a pronounced effect on oscillatory activity during REM sleep, while NREM sleep oscillations were less affected. REM sleep has been implicated in the integration and stabilization of memory traces, complementing the role of NREM sleep in initial memory consolidation and hippocampal–cortical communication [11,44]. The selective disruption of REM-related oscillations in *Limk1* KO mice suggests that LIMK1 may contribute not only to the early stages of memory consolidation during NREM sleep but also to later processing and integration of memories during REM sleep. This finding highlights the potential role of LIMK1 in coordinating sleep stage-specific processes that collectively support long-term memory formation, and warrants further investigation to dissect the mechanisms by which LIMK1 regulates REM-associated plasticity and memory integration.

To further confirm whether LIMK1 loss affects interregional dialogue crucial for memory consolidation, we implanted electrodes in the hippocampus and prefrontal cortex and observed changes in the properties of characteristic oscillations in different brain regions, including energy distribution and peak differences (Figure 3A–K). Previous research has reported that neural oscillation coupling between the hippocampus and prefrontal cortex affects memory consolidation and that structural and functional changes in neurons can disrupt interregional dialogue, ultimately impairing memory consolidation [8,45,46,47,48]. Therefore, we conducted relevant tests in LIMK1 knockout mice, and the results showed that coupling exhibited an increased frequency after memory encoding in normal mice, while this change was not observed in knockout mice (Figure 3K). Based on this, we speculate that the loss of LIMK1 likely affects neuronal plasticity changes, thereby interfering with information transmission between the two brain regions and ultimately impairing memory consolidation.

Based on the previous results, it is evident that LIMK plays a crucial role in memory consolidation [21,49,50]. In certain neurological disorders, excessive consolidation of memory is one of the mechanisms underlying neural abnormalities [26,28,51,52]. Therefore, based on previous research, we established an underwater traumatic stress mouse model [32,53]. After exposure to underwater trauma, mice displayed significant anxiety phenotypes in the water-associated zero maze (Figure 4A–E). To investigate whether LIMK1 plays a critical role in this process, we examined the phosphorylation levels of LIMK1 and cofilin by Western blotting to evaluate changes in their signaling activity (Figure 4F–N). The results revealed that both LIMK1 and cofilin were activated following trauma exposure; however, the activation timing differed markedly between brain regions. Specifically, LIMK1 and cofilin phosphorylation increased in the hippocampus approximately 2 h after trauma, whereas similar activation occurred in the mPFC at around 6 h post-trauma.

This temporal sequence suggests a hippocampus to prefrontal cortex cascade, consistent with the established model of memory consolidation in which newly encoded information is initially processed within the hippocampus and subsequently transferred to cortical areas for long-term storage [54,55,56]. The delayed phosphorylation in the mPFC may thus reflect downstream plasticity events driven by earlier hippocampal activation [57]. Such sequential activation dynamics indicate that LIMK1 mediated actin cytoskeletal remodeling may participate in both early encoding-related plasticity within the hippocampus and later systems-level consolidation in the prefrontal cortex [17,58]. Further investigation using time-resolved manipulation of LIMK1 signaling could help determine whether this temporal pattern represents a causal mechanism for hippocampal–cortical information transfer and memory stabilization [59].

To verify the impact of LIMK1 function on trauma memory formation, we administered LIMK inhibitors via cannulation to inhibit LIMK activity in different brain regions. The results demonstrated that a single hippocampal or mPFC injection of the inhibitor significantly reduced the incidence of anxiety behavior (Figure 5). This indicates that a single administration of the LIMK inhibitor is sufficient to suppress stress-induced LIMK1 activity changes in the hippocampus-cortical circuit. Based on these results, we can infer that intervention targeting LIMK1 to modulate the functional activity of memory consolidation-related brain regions can effectively ameliorate memory abnormalities in post-traumatic stress disorder (PTSD). Importantly, while our results link LIMK1 activation to Cofilin phosphorylation, we have not yet directly tested whether LIMK1 inhibition prevents Cofilin phosphorylation in the UWT model, an important question for future studies aimed at clarifying the mechanistic role of LIMK1 in traumatic memory formation.

Despite its potential, targeting LIMK1 presents several challenges. First, the commonly used inhibitor LIMK-i3 also affects LIMK2, another LIMK family member involved in neuronal function, so its effects may reflect combined inhibition of both kinases. Additionally, LIMK-i3 has a relatively large molecular weight, which hinders its ability to efficiently cross the blood–brain barrier when administered orally or via injection [60,61]. Therefore, more specific and brain-permeable LIMK1 inhibitors are needed to precisely evaluate its therapeutic potential. In this study, we employed a single injection to transiently inhibit LIMK1 activity, with the dosage based on prior research [21]. However, early intervention at the moment of trauma is often impractical in clinical settings [62]. Future studies should therefore assess the efficacy of LIMK1 inhibition at later post-encoding phases, which would better reflect real-world therapeutic scenarios. Moreover, potential adverse effects must also be considered, as LIMK1 inhibition could disrupt the consolidation of other adaptive or beneficial memories [63,64]. Species differences further complicate translational interpretation, as humans and mice differ markedly in the temporal dynamics and architecture of sleep, a key process in memory consolidation [1,10,65,66,67]. Further investigations are needed to determine the optimal dose and administration frequency for effectively targeting memory consolidation after traumatic stimulation. Furthermore, due to the limitations of electrode implantation, the electrodes became wet during the underwater process. As a result, we were unable to collect neural oscillations from different brain regions following underwater trauma, which prevented us from performing coupling-related analyses. Lastly, other brain regions, such as the amygdala and bed nucleus of the stria terminalis (BNST), also play critical roles in the consolidation of traumatic memories [68,69,70]. However, we focused solely on the function of LIMK1 in the hippocampus-mPFC circuit here, while the roles of other brain regions will need to be explored in future studies.

In summary, this study confirms the crucial role of LIMK1 in memory consolidation. Furthermore, we compared the sleep structure and found no significant differences in knockout mice, but notable differences were observed in brainwave activity during different states. By synchronously recording the hippocampus and mPFC in vivo, we revealed that the absence of LIMK1 affects interregional coupling dialogue, thereby influencing memory consolidation. In the context of abnormal memory consolidation in PTSD, inhibiting LIMK1 activity with inhibitors can ameliorate the pathological phenotypes of PTSD. These results indicate the important role of LIMK1 in memory processes and suggest that it can be targeted to modulate the excessive consolidation of memory in related psychiatric disorders.

## 5. Conclusions

Our study demonstrates that LIMK1 is a key regulator of memory consolidation in the hippocampus and prefrontal cortex. Both genetic deletion and pharmacological inhibition of LIMK1 impaired long-term, but not short-term, memory formation in mice. Although sleep architecture was unchanged in knockout animals, alterations in brainwave activity during slow-wave and rapid eye movement sleep suggested disrupted neural dynamics. Electrophysiological recordings further revealed that LIMK1 deficiency weakened hippocampal–prefrontal coupling during the consolidation phase, highlighting its essential role in maintaining interregional communication required for stable memory storage. In a trauma stress model, region-specific activation of LIMK1 was observed after exposure, and pharmacological inhibition in the hippocampus effectively reduced PTSD-like behaviors, whereas prefrontal inhibition had no similar effect. These results indicate that LIMK1-dependent signaling contributes to both physiological and pathological memory consolidation and may serve as a potential therapeutic target for trauma-related disorders. However, current LIMK inhibitors lack specificity and brain permeability, emphasizing the need for improved compounds and circuit-level investigations to fully elucidate LIMK1′s role in abnormal memory processes.

## Data Availability

The data that support the findings of this study are available from the corresponding author upon reasonable request.

## Supplementary Materials

The following supporting information can be downloaded at: https://www.mdpi.com/article/10.3390/biology14111560/s1, Figure S1: Effects of LIMK1 deficiency or functional inhibition on mouse locomotion.; Figure S2: Raw co-occurrence of brain rhythms before and after memory encoding.; Figure S3: Expression levels of LIMK1 and Cofilin in the hippocampus and mPFC at different time points after UWT modeling.
