# Supplementary material for: LIMK1 Deficiency Disrupts Hippocampal–Cortical Memory Consolidation and Attenuates Trauma-Induced PTSD-like Behavior
Source: Biology (Basel). 2025 Nov 7;14(11):1560. doi: 10.3390/biology14111560 (PMC12650199; doi:10.3390/biology14111560)
Supplement: Supplementary file 1 [file biology-14-01560-s001.zip › biology-3898489-supplementary.pdf]

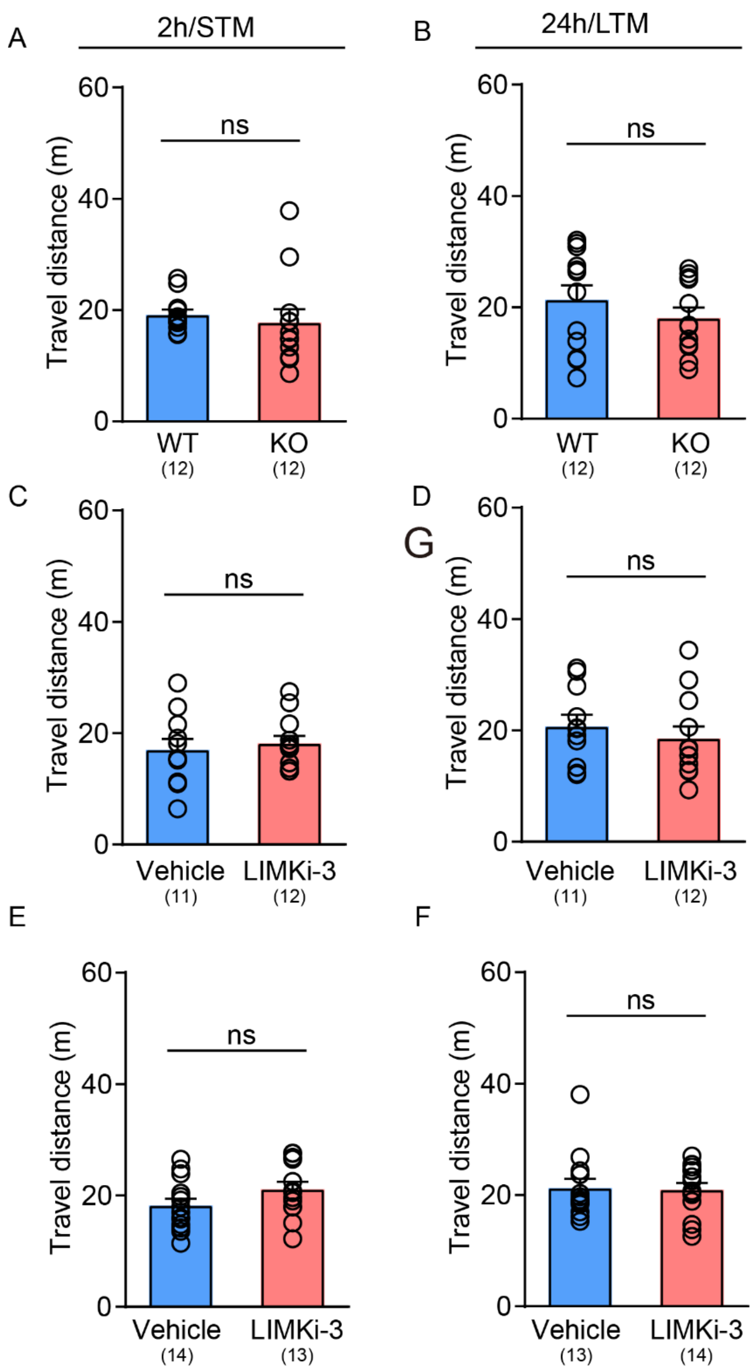

Supplementary Figure S1. Effects of LIMK1 deficiency or functional inhibition on mouse locomotion. A, B. LIMK1 knockout does not affect mouse locomotion during the OLR behavioral test. C, D. Injection in the mPFC region does not influence locomotion. E, F. Injection in the hippocampus similarly shows no significant effect on locomotion. Data are presented as mean ± SEM. Statistical analysis was performed using Student's t-test. *n* for each group is provided in the figure. ns: Not significant.

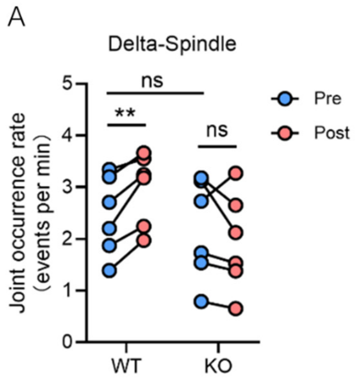

Supplementary Figure S2. Raw co-occurrence of brain rhythms before and after memory encoding. Non-normalized co-occurrence values of hippocampal–cortical oscillatory events (A. Delta–spindle, B. Ripple–delta, and C. Ripple–delta–spindle couplings) are shown for pre-encoding and post-encoding periods in both WT and *Limk1* KO mice. Data represent the raw baseline and post-encoding co-occurrence levels used for normalization in Figure 3, allowing direct comparison of baseline rhythmic coupling between groups. Paired Student's t-test was used for pre- vs. post-encoding comparisons, and unpaired Student's t-test for WT vs. KO comparisons. n = 6 per group. *$p < 0.05$, **$p < 0.01$, ns: Not significant.

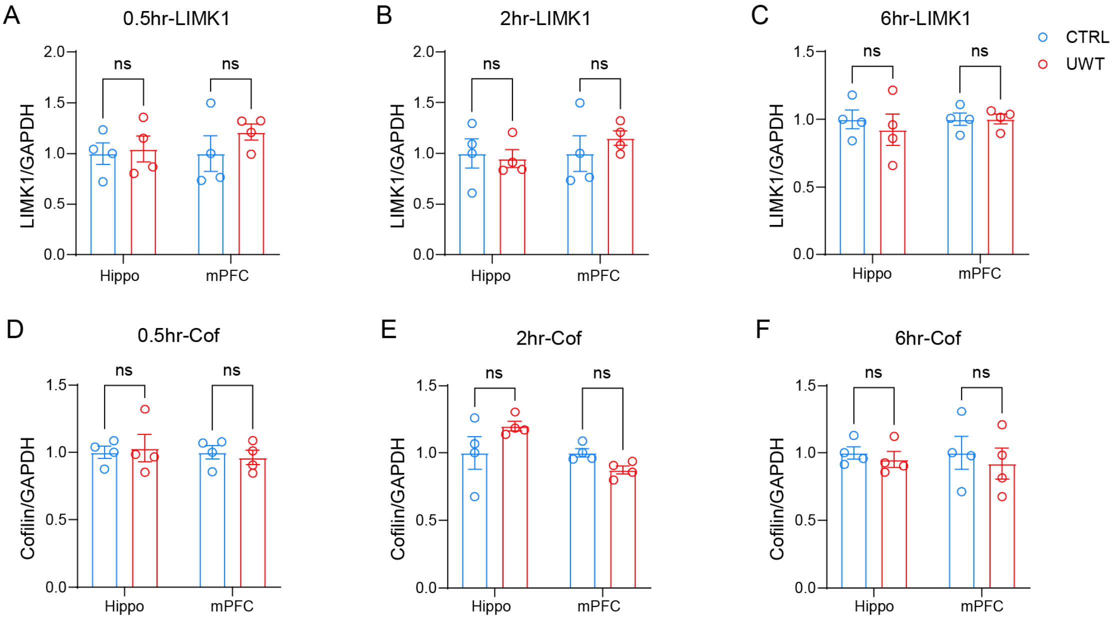

Supplementary Figure S3. Expression levels of LIMK1 and Cofilin in the hippocampus and mPFC at different time points after UWT modeling. A, B, C. Expression levels of LIMK1 in the hippocampus and prefrontal cortex at 0.5 h, 2 h, and 6 h after UWT modeling; D, E, F. Expression levels of Cofilin in the hippocampus and prefrontal cortex at 0.5 h, 2 h, and 6 h after UWT modeling. Data are presented as mean ± SEM. Statistical analysis was performed using Student's t-test. $n = 4$ for each group. ns: Not significant.
